# An exploratory analysis of the competing effects of alcohol use and advanced hepatic fibrosis on serum HDL

**DOI:** 10.1007/s10238-021-00736-6

**Published:** 2021-07-01

**Authors:** Augustin G. L. Vannier, Amanda PeBenito, Vladislav Fomin, Raymond T. Chung, Esperance Schaefer, Russell P. Goodman, Jay Luther

**Affiliations:** 1 Division of Gastroenterology, MGH Alcohol Liver Center, Massachusetts General Hospital, Harvard Medical School, Boston, MA 02114 USA; 2 Department of Medicine, Gastrointestinal Unit, Massachusetts General Hospital, Harvard Medical School, Boston, MA 02114 USA; 3 Department of Medicine, Massachusetts General Hospital, Harvard Medical School, Boston, MA 02114 USA

**Keywords:** Alcohol-associated liver disease, Alcohol use, HDL

## Abstract

While alcohol use has been shown to increase serum HDL, advanced liver disease associates with decreased serum HDL. The combined influence of alcohol consumption and liver fibrosis is poorly defined. In this study, we sought to investigate the competing effects of alcohol use and hepatic fibrosis on serum HDL and to determine if the presence of advanced hepatic fibrosis ablates the reported effect of alcohol consumption on serum HDL. We performed a cross-sectional, exploratory analysis examining the interaction between alcohol use and advanced hepatic fibrosis on serum HDL levels in 10,528 patients from the Partners Biobank. Hepatic fibrosis was assessed using the FIB-4 index. We excluded patients with baseline characteristics that affect serum HDL, independent of alcohol use or the presence or advanced hepatic fibrosis. We observed an incremental correlation between increasing HDL levels and amount of alcohol consumed (*P* < 0.0001), plateauing in those individuals who drink 1–2 drinks per day, Contrastingly, we found a negative association between the presence of advanced hepatic fibrosis and lower HDL levels, independent of alcohol use (beta coefficient: -0.011075, SEM0.003091, *P* value: 0.0001). Finally, when comparing subjects with advanced hepatic fibrosis who do not use alcohol to those who do, we observed that alcohol use is associated with increased HDL levels (54.58 mg/dL vs 67.26 mg/dL, *p* = 0.0009). This HDL-elevating effect of alcohol was more pronounced than that seen in patients without evidence of advanced hepatic fibrosis (60.88 mg/dL vs 67.93 mg/dL, *p* < 0.0001). Our data suggest that the presence of advanced hepatic fibrosis does not blunt the HDL-elevating effect of alcohol use.

## Introduction

Excessive alcohol use is a frequent cause of morbidity and mortality worldwide. In fact, alcohol use is one of the most common causes of deaths and disability-associated life years (DALYs) [1], as it is mechanistically linked with the development of multiple diseases. Most notably, Alcohol-associated Liver Disease (ALD) is one of the most common manifestations of alcohol misuse and accounts for close to 1/3rd of all alcohol-related deaths [1]. Interestingly, multiple observational studies have demonstrated a positive association between moderate alcohol use and high-density lipoprotein (HDL) levels [2–6].

The regulation of serum HDL, a cholesterol subtype, is complex. The liver accounts for a majority of the total serum HDL levels and is intimately involved in all aspects of HDL regulation, from synthesis to transport to metabolism. Accordingly, previous studies have demonstrated reduced HDL levels in patients with both acute and chronic liver diseases [7–9]. Additionally, data suggest an inverse association between HDL levels and hepatic function. Specifically, reduced serum HDL levels have been associated with severity of liver disease, the presence of liver-related complications and mortality in patients with cirrhosis [10].

Despite the observed associations of alcohol and liver disease with serum HDL, there are limited data examining the competing effects of each in a well-characterized cohort. To address this question, we investigated the interaction between alcohol use and the presence of advanced hepatic fibrosis in a large cohort of patients unaffected by major sources of HDL variation: hepatitis B or C infection, primary biliary cholangitis, poor glycemic control, obesity, heavy smoking or lipid-lowering medications. Thus, we sought to isolate the combined effects of alcohol and fibrosis on serum HDL.

## METHODS

### Clinical database

Clinical data were obtained from the Partners Biobank [11], which is a large integrated database from Mass General Brigham healthcare, comprised of approximately 121,691 consented subjects with variable clinical data that is available. Within the Mass Hospital Brigham system, there is an online platform which allows for a detailed search of this cohort to allow identification of patients that have available data that fits the proposed clinical question. We queried this database to identify patients that met inclusion criteria, which are detailed below. These criteria were determined a priori. Data were derived from electronic health records, which include patient data since 1990. Informed consent was obtained from all study participants and/or their guardians.

### Patient selection

Patients were included in the study if they had all of the following available for analysis: 1) lipid panels, 2) fully completed alcohol questionnaires; and 3) clinical data to allow for noninvasive assessment of hepatic fibrosis. Once patients were identified, we pulled demographics, medication, laboratory and alcohol use data. The most recent laboratory data available in the dataset were used for analysis. The date of questionnaire completion is not recorded in the Biobank dataset; therefore, we were unable to ascertain the timing between questionnaire completion and laboratory evaluation. The alcohol questionnaire administered for this study asked the following question: “During the past year, how many alcoholic drinks (glass/bottle/can of beer; 4 oz glass of wine; drink or shot of liquor) did you usually drink in a typical week?”. Participants were given the following options: 1) None, or less than one per month, 2) 1–3 per month, 3) 2–4 per week, 4) 5–6 per week, 5) 1–2 per day, 6) 3–4 per day, 7) 5–6 per day, or 9) more than 6 per day.

We excluded variables which have been shown to affect serum HDL levels, in order to isolate the effects of alcohol and fibrosis on HDL. Therefore, patients were excluded if they had obesity (BMI ≥ 30), poor glycemic control (Hemoglobin A1C > 6.5), a smoking history greater than 75 pack-years, or used lipid-lowering medications. In addition, we excluded patients with a liver disease known to affect HDL independently of fibrosis. We therefore excluded patients with primary biliary cholangitis or evidence of prior or active Hepatitis B or C infection [12–15].

### Assessment of hepatic fibrosis

The Fibrosis-4 Index (FIB-4) was used to determine the presence of advanced hepatic fibrosis. The FIB-4 was calculated using the following formula: (Age [years] * AST)/(Platelet count[10 × 109]*√(ALT).

Patients withFIB-4 score of greater than or equal to 3.25 were defined as having advanced hepatic fibrosis, as this cut-off value has been shown to have high diagnostic accuracy for advanced hepatic fibrosis in patients with ALD[16]. This value has been associated with METAVIR, Batts and Ludwig, or Scheuer stages F3 through F4 or Ishak stages F4 through F6. [17].

### Statistical analyses

Comparisons between two groups were performed with unpaired, two tailed, Welch t tests and comparisons among three or more groups were performed with one-way ANOVA with follow-up Tukey tests. Comparison of categorical variables was performed with Fisher’s exact test. Risk factors for severe liver disease were examined with the use of univariate and multivariate logistic regression; all factors in the univariate analysis were included in the multivariate analysis. A beta coefficient value was calculated to measure the degree to which each variable influenced the severity of liver disease, with *P* < 0.05 considered significant. All analyses were conducted using GraphPad Prism (San Diego, CA).

## Results

### Demographics

**Fig. 1:**
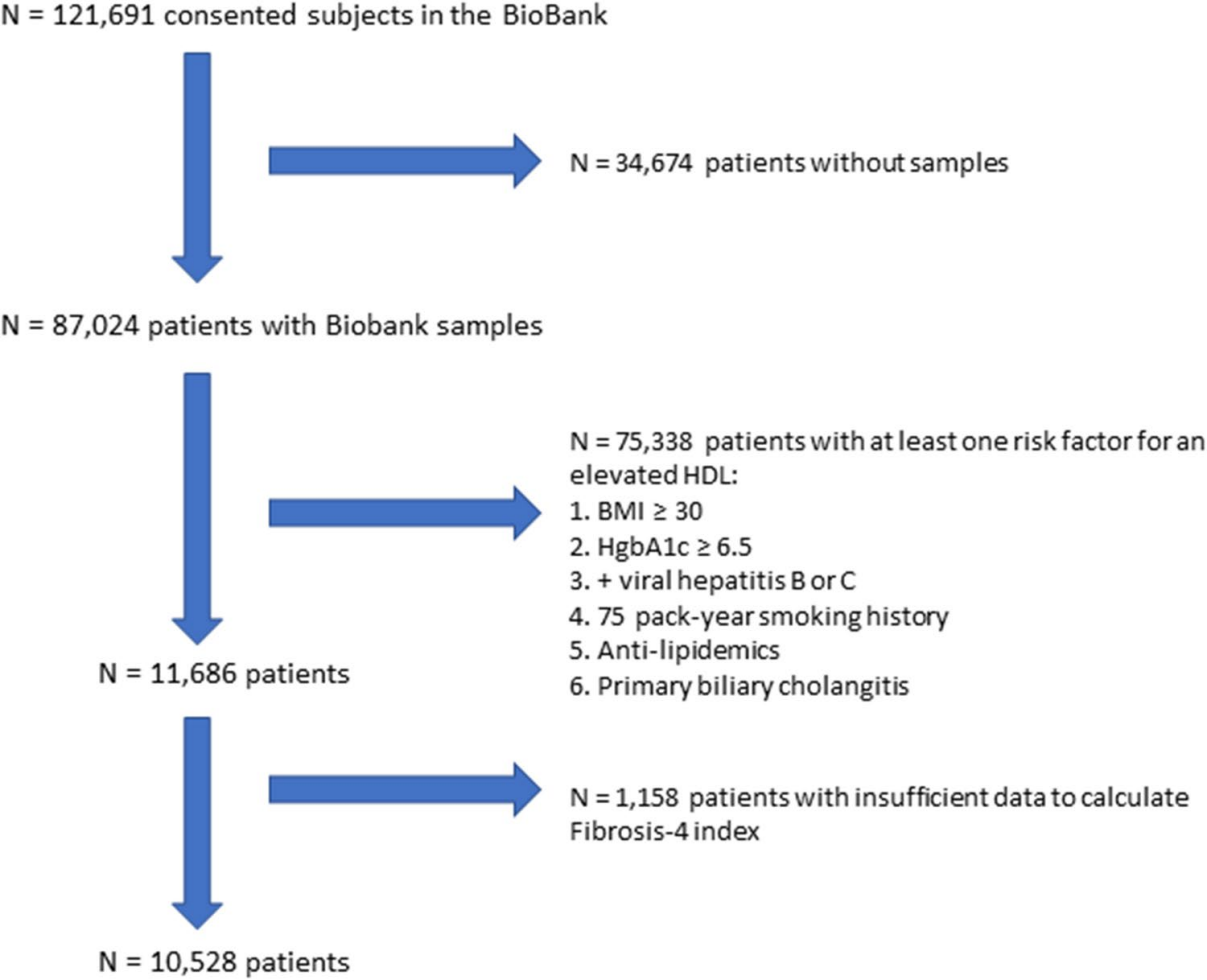
Flow diagram of patient selection. The flow diagram details the method for patient selection in our analyses

The total cohort of patients included in this analysis consisted of 10,528 patients (Fig. 1, Table 1). The majority of patients studied were middle-age (52.4 years), female (70.2%) and white (93.5%). As noted previously, patients were not obese and had no history of significant insulin resistance or smoking. Approximately one-half of the patients included in the analysis drank either 0–1 drinks per month or 2–4 drinks per week, with a minority of patients (3.5%) drinking 3 or more alcohol beverages per day.

**Table 1:** Patient demographics

	Total
N	10,528
Age	52.4
Sex (%Female)	70.2
BMI	24.0
HgbA1c	5.3
LDL	103.9
Triglycerides	97.9
Race/Ethnicity	
White (%)	93.5
Asian (%)	3.8
Black (%)	2.7
Hispanic (%)	1.8

Alcohol use N (%)	
0–1 /month	2565 (24.4)
1–3 /month	1422 (13.5)
1 /week	969 (9.2)
2–4 /week	2426 (23)
5–6 /week	1627 (15.5)
1–2 /day	1155 (11)
3–4 /day	280 (2.7)
5–6 /day	47 (0.4)
> 6 /day	37 (0.4)

N = number; BMI = body mass index; HgbA1c = hemoglobin A1c; LDL: low-density lipoprotein

With regards to liver disease severity, 373 patients had serologic evidence of advanced hepatic fibrosis while 10,155 subjects did not. These populations differed in multiple ways (Table 2), including age (mean age 58.9 versus 52). The cohort of patients with available data to study liver disease severity had more women than men (70% female) and while this distribution was also observed in the group without liver disease, there was a higher representation of males in the fibrosis group (53.2% female). The two groups in our hepatic fibrosis analyses were similar in terms of their BMI and racial and ethnic distributions.

**Table 2:** Patient demographics based on the presence or absence of hepatic fibrosis

	No fibrosis	Fibrosis	*P* value
N	10,032	496	
Age	52.0	58.9	< 0.0001
Sex (%Female)	71.0	53.2	< 0.0001
BMI	24.0	24.0	0.86
Hgb A1c	5.3	5.3	0.37
LDL	103.9	103.1	0.62
Triglycerides	97.4	109.0	< 0.0001
Race/Ethnicity			1.0
White (%)	93.4	94.2	
Asian (%)	3.9	3.0	
Black (%)	2.7	2.8	
Hispanic (%)	1.8	2.3	

N = number; BMI = body mass index; HgbA1c = hemoglobin A1c; LDL: low-density lipoprotein

### Incremental increase in serum HDL levels associated with alcohol use

**Fig. 2:**
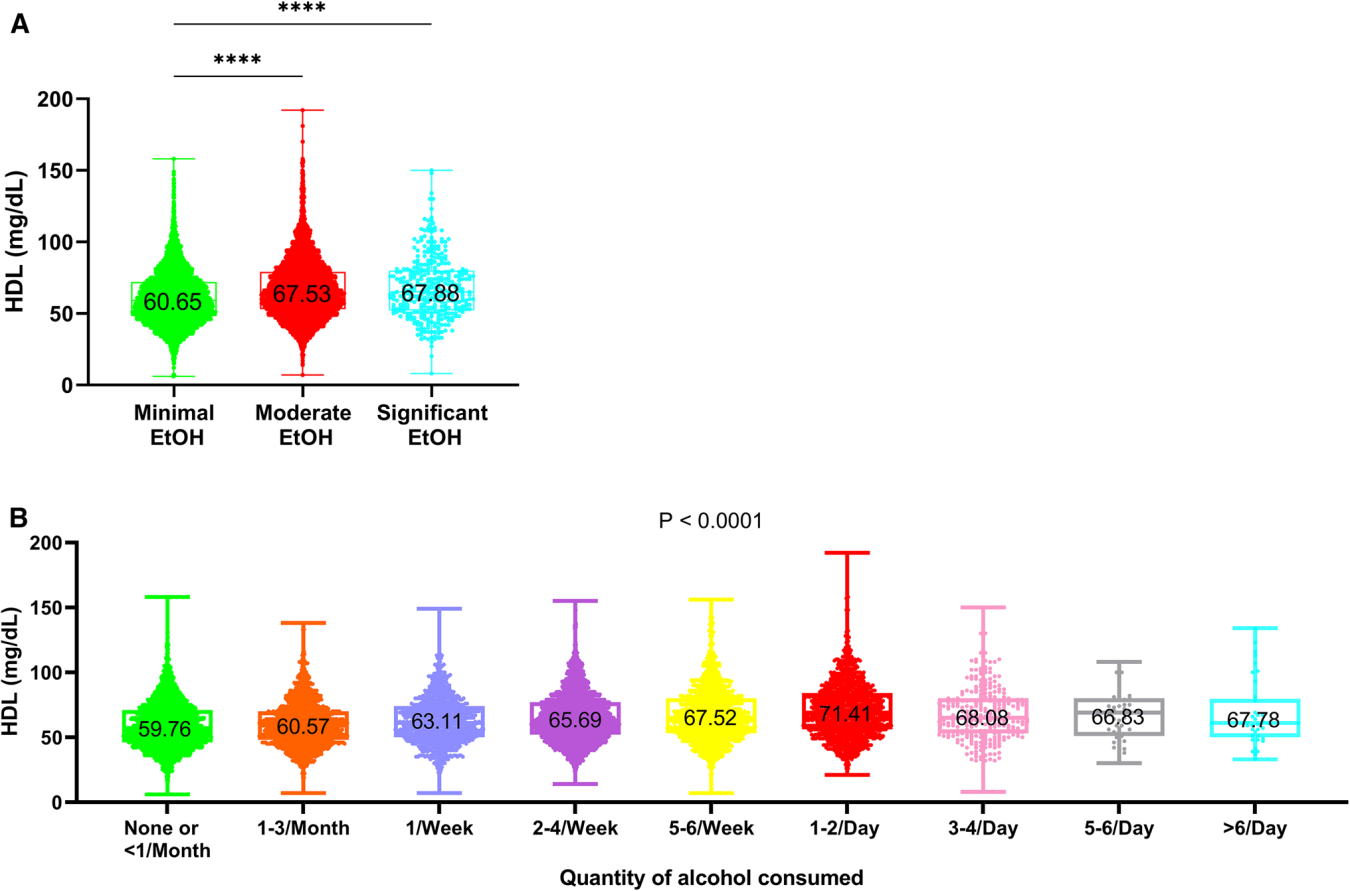
Increasing use of alcohol associated with elevated serum HDL levels. We analyzed the relationship between alcohol drink quantity and serum HDL levels in our cohort of 10,528 patients. (**a**) A box and whiskers plot showing the association of quantity of alcohol consumed with HDL levels. The number within box represents the average serum HDL in that group. (**b**) A box and whiskers plot demonstrating the relationship between minimal (0–1 drinks/week), moderate (2–14 drinks per week) and significant (3–6 drinks per day) alcohol use and HDL. HDL = high-density lipoprotein; EtOH = alcohol. **** *P* < 0.0001

Previous data have suggested a positive correlation between alcohol use and serum HDL levels [2–4]. To investigate this in our cohort, we examined the association of HDL levels with quantity of alcohol consumed. While patients that abstained from alcohol or had less than one drink per month (*n* = 2565) had an average HDL of 59.76 mg/dL, those that drank one or more alcoholic drink daily (*n* = 1519) had an average HDL of 70.56 mg/dL (*P* < 0.0001). Similarly, we found a difference in HDL levels in those patients with severe (3–6 drinks per day), moderate (2–14 drinks/week) and minimal (0–1 drinks/week) alcohol consumption (*P* < 0.0001) (Fig. 2a). Furthermore, we found that HDL levels steadily rose incrementally with drink quantity, plateauing in those individuals who drink 1–2 drinks per day (Fig. 2b). Taken together, these data suggest a positive association between alcohol use and serum HDL levels.

### Advanced hepatic fibrosis associates with reduced serum HDL levels

**Fig. 3:**
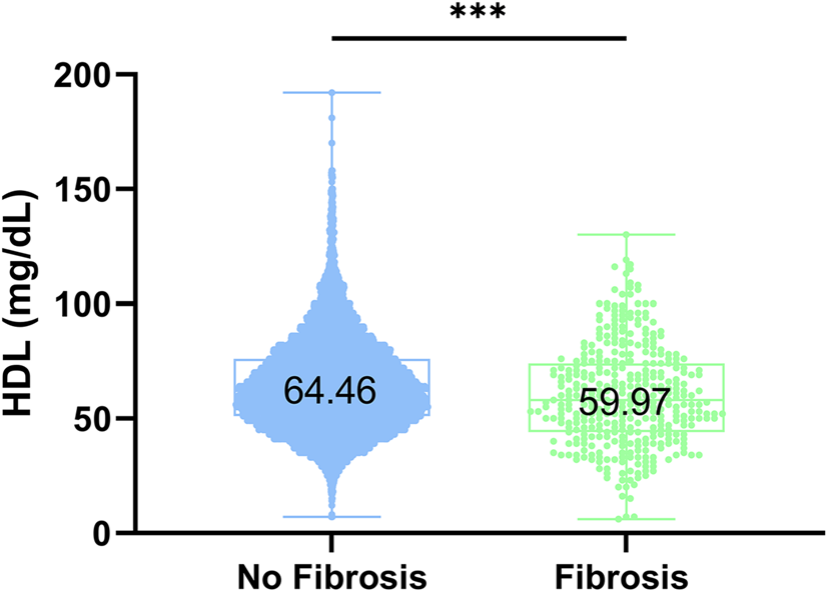
Increasing liver disease severity associates with reduced serum HDL levels. A box and whiskers plot demonstrating the relationship between the presence of advanced or absence of hepatic fibrosis, based on the Fibrosis-4 index and serum HDL levels. HDL = high-density lipoprotein. *** *P* = 0.0001

We next sought to examine whether the presence of advanced hepatic fibrosis was associated with serum HDL levels in our cohort. To investigate this, we defined the presence of advanced hepatic fibrosis as those patients with a FIB-4 score of ≥ 3.25. In the 373 patients with evidence of advanced hepatic fibrosis, HDL levels were lower compared to the 10,155 subjects without evidence of advanced hepatic fibrosis (Fig. 3) (mean HDL 59.97 mg/dL vs. 64.46 mg/dL, *p* < 0.0001). Furthermore, in a univariate analysis, we found that HDL was associated significantly with liver disease severity, as did age and gender in our cohort (Table 3). In a multivariate analysis accounting for age and gender, we found that the association between HDL levels and advanced hepatic fibrosis maintained significance (Beta coefficient: -0.01175, SEM: 0.003091, *P* value: 0.0001). Taken together, these data support the negative association between the presence of advanced hepatic fibrosis and serum HDL levels.

**Table 3:** Results for univariate analysis for factors influencing liver disease severity

Variable	Beta coefficient	SEM	*P* Value
HDL	− 0.1248	0.002896	< 0.0001
Age	0.04918	0.003666	< 0.0001
Gender	− 0.7614	0.1060	< 0.0001
Race	− 0.2679	0.1995	0.1793

SEM = standard error of the mean

### The combined effect of alcohol use and advanced hepatic fibrosis on serum HDL levels

**Fig. 4:**
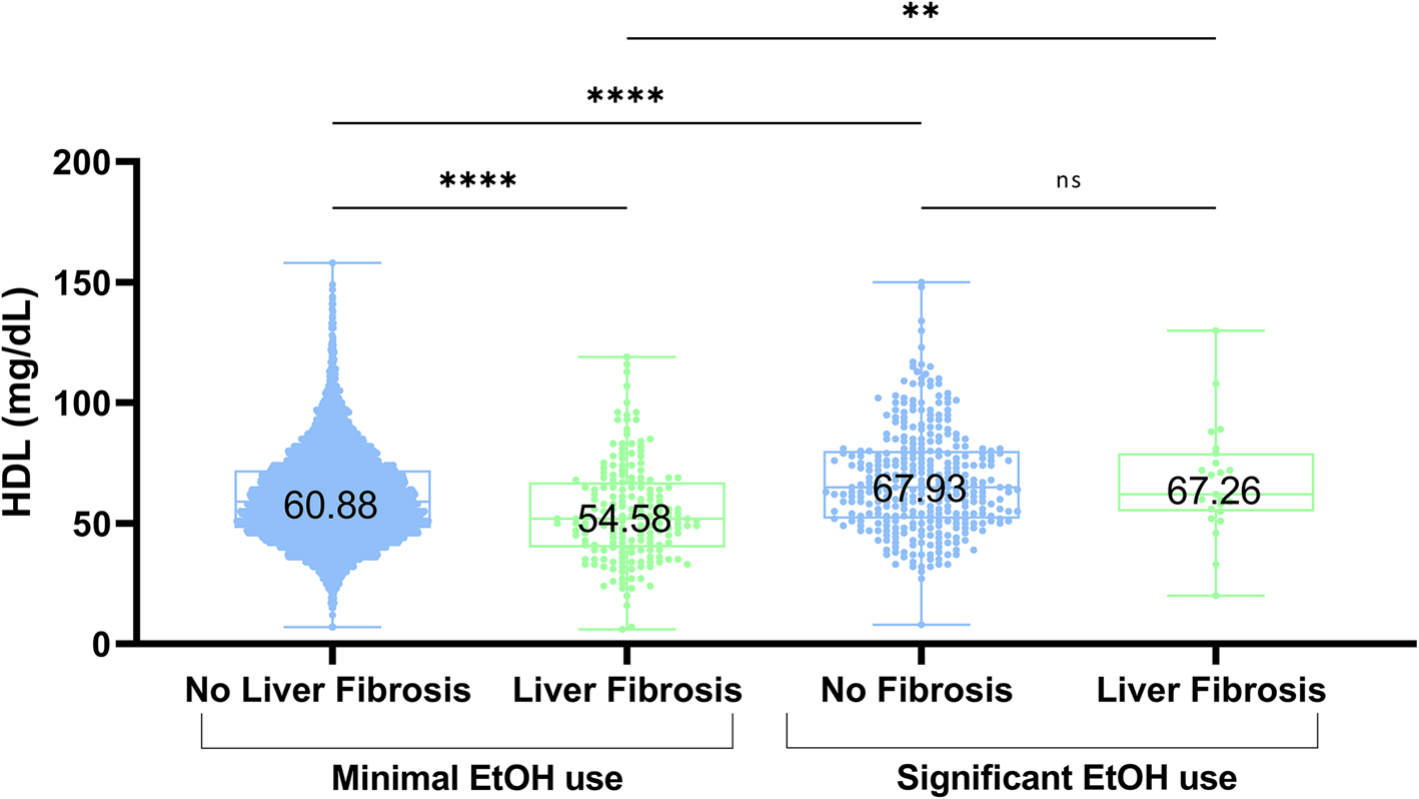
Increasing alcohol use associates with elevated serum HDL, even patients with evidence of hepatic fibrosis. A box and whiskers plot illustrating serum HDL levels in patients with a varying level of liver disease, based on the Fibrosis-4 index and alcohol use patterns (minimal: 0–1 drinks per week) and significant: 3–6 drinks per day). ** *P* = 0.001; **** *P* < 0.0001; ns = not significant

Given the observed competing effects of alcohol and advanced hepatic fibrosis on serum HDL levels in our cohort, we next determined the combined effect of these variables on serum HDL. To accomplish this, we stratified subjects by both alcohol use and the presence or absence of advanced hepatic fibrosis. In subjects with minimal alcohol use, defined as less than 1 drink per week (0–3 per month), those with advanced hepatic fibrosis have significantly lower HDL levels compared to those patients without advanced fibrosis (Fig. 4). However, when examining patients with significant alcohol use (3 or more drinks per day) this relationship was lost and both patients with and without advanced hepatic fibrosis had comparable HDL levels (67.26 mg/dL vs 67.93 mg/dL, *p* = 0.9997). Likewise, when comparing subjects with advanced hepatic fibrosis who do not use alcohol to those who do, we again observed that alcohol use is associated with increased HDL levels, even in the setting of fibrosis (54.58 mg/dL vs 67.263 mg/dL, *p* = 0.0078). This HDL-elevating effect of alcohol was comparable and even greater than that seen in patients without advanced hepatic fibrosis (60.88 mg/dL vs 67.93 mg/dL, *p* < 0.0001). Taken together, these data suggest that while liver disease is associated with lower HDL levels, it does not blunt the HDL-elevating effect of alcohol use.

## Discussion

In our exploratory analysis of a large cohort of patients, we reaffirmed the findings that heavier alcohol use associates with increased HDL levels and that the presence of advanced hepatic fibrosis associates with reduced serum HDL levels. In addition, our analysis highlights the fact that the HDL-elevating effect of alcohol is maintained in those patients with evidence for advanced hepatic fibrosis.

While multiple previous studies have documented the effects of alcohol and liver disease on serum HDL, there are limited and remote data examining their competing effects in the same cohort [18, 19]. In our exploratory analysis, we found that the ability of alcohol to raise HDL levels occurred despite the presence of advanced hepatic fibrosis. While there may be multiple biological explanations for this finding, our results raise the possibility that the effect of alcohol on serum HDL is regulated in a liver-independent manner. The critical role of the liver in regulation of HDL is not debated. Decades of mechanistic and epidemiological studies have demonstrated the importance of preserved hepatic functioning in all aspects of HDL regulation, from synthesis to transport to metabolism [20]. For example, previous translational work has suggested that alcohol increases the transport rates of apoA-I and apoA-II, two major HDL lipoproteins [21]. Mechanistic work has also identified the effect of alcohol on reverse cholesterol transport, mediated in part by PPAR-γ, which impacts serum HDL levels [22]. Additionally, certain data suggest an inhibitory effect of alcohol on the activity of cholesteryl-ester transfer protein (CEPT), a key transporter of cholesterol from HDL to LDL, leading to higher HDL levels [23]. These data reinforce the importance of preserved hepatic function in the regulation of HDL. Patients with advanced hepatic fibrosis have reduced liver function and are therefore expected to have a reduced ability to modulate HDL in a liver-dependent manner in response to alcohol. However, we did not find this in our study. Because patients with advanced hepatic fibrosis and active alcohol use had significantly higher serum HDL levels compared to those with advanced hepatic fibrosis who were not drinking, it is possible that novel and liver-independent pathways also contribute to alcohol’s effect on serum HDL. Further mechanistic work is needed to better understand this relationship.

The association of alcohol use with increased HDL levels is of unclear clinical impact. Several studies have found that low to moderate alcohol consumption is associated with improved cardiovascular-related survival compared with abstainers and heavy drinkers [1, 24, 25] and that this observed association may be related to elevated HDL levels in patients with alcohol use. However, some studies challenge this hypothesis. In particular, Mendelian randomization studies have shown that genetic variants which naturally increase HDL levels do not confer a cardioprotective benefit [26] and that genetic variants linked with non-drinking or low amounts of alcohol consumption are associated with favorable cardiovascular profiles [27]. Similarly, there is little evidence that pharmacotherapy directly targeting HDL is associated with improved cardiovascular outcomes. Further studies examining the effect of elevated HDL levels in patients with alcohol use and liver disease on cardiovascular outcomes are warranted.

Despite our findings, certain limitations of our exploratory analysis must be highlighted. First, we relied on FIB-4 scores as a marker of the presence of hepatic fibrosis. While this marker has been validated and used in numerous epidemiological studies [28–30], it is not the gold standard for hepatic fibrosis assessment and was originally validated in liver disease not caused by alcohol. However, the FIB-4 index was shown to have diagnostic accuracy for identifying advanced hepatic fibrosis in patients with ALD [16]. Second, we were unable to determine the interval time between completion of the alcohol questionnaire and laboratory evaluation. It is therefore possible that patients meaningfully changed their alcohol consumption habits from the time of completing the questionnaire to when we evaluated HDL levels, which may have affected the results. Finally, the vast majority of patients in our analysis were white and female, limiting the ability to generalize our results and raising the possibility of selection bias. Additionally, our observed prevalence of advanced hepatic fibrosis (~ 4%) is less than what is reported in the literature [31], further amplifying the possibility of selection bias.

In summary, our exploratory investigation into the effects of alcohol and liver disease on HDL levels suggest that the presence of liver disease does not blunt the effect of alcohol on HDL, raising the possibility that the alcohol elevating effect on HDL occurs independently of preserved liver function.
